# Metabarcoding on both environmental DNA and RNA highlights differences between fungal communities sampled in different habitats

**DOI:** 10.1371/journal.pone.0244682

**Published:** 2020-12-30

**Authors:** Martino Adamo, Samuele Voyron, Matteo Chialva, Roland Marmeisse, Mariangela Girlanda

**Affiliations:** 1 Univ Lyon, Université Claude Bernard Lyon 1, CNRS, INRAE, VetAgro Sup, UMR Ecologie Microbienne, Villeurbanne, France; 2 Dipartimento di Scienze della Vita e Biologia dei Sistemi, Università degli Studi di Torino, Torino, Italy; 3 Istututo di Protezione delle Piante, Consiglio Nazionale delle Ricerche, Torino, Italy; 4 Institut de Systématique, Évolution, Biodiversité (ISYEB), Muséum national d’histoire naturelle, CNRS, Sorbonne Université, EPHE, Université des Antilles, Paris, France; University of Hyogo, JAPAN

## Abstract

In recent years, metabarcoding has become a key tool to describe microbial communities from natural and artificial environments. Thanks to its high throughput nature, metabarcoding efficiently explores microbial biodiversity under different conditions. It can be performed on environmental (e)DNA to describe so-called total microbial community, or from environmental (e)RNA to describe active microbial community. As opposed to total microbial communities, active ones exclude dead or dormant organisms. For what concerns Fungi, which are mostly filamentous microorganisms, the relationship between DNA-based (total) and RNA-based (active) communities is unclear. In the present study, we evaluated the consequences of performing metabarcoding on both soil and wood-extracted eDNA and eRNA to delineate molecular operational taxonomic units (MOTUs) and differentiate fungal communities according to the environment they originate from. DNA and RNA-based communities differed not only in their taxonomic composition, but also in the relative abundances of several functional guilds. From a taxonomic perspective, we showed that several higher taxa are globally more represented in either “active” or “total” microbial communities. We also observed that delineation of MOTUs based on their co-occurrence among DNA and RNA sequences highlighted differences between the studied habitats that were overlooked when all MOTUs were considered, including those identified exclusively by eDNA sequences. We conclude that metabarcoding on eRNA provides original functional information on the specific roles of several taxonomic or functional groups that would not have been revealed using eDNA alone.

## Introduction

Metabarcoding, i.e. the combined use of universal DNA barcodes and high-throughput sequencing, is now a standard approach to characterize microbial communities from nucleic acids directly extracted from environmental samples (soil, plants, sediment, fresh or sea waters) [1]. This strategy is widely used to assess biodiversity and how it is impacted by anthropogenic disturbance and other environmental factors [2]. In the case of Fungi, metabarcoding complements or can substitute traditional ecosystem biomonitoring protocols often based on the collection and expert identification of individual species [3, 4]. Metabarcoding also allows identification of the numerous fungal species that are not cultivable [2, 5], or overlooked during traditional field surveys.

At the global scale, the monitoring of fungal diversity by metabarcoding has shown how it is shaped by a wide set of environmental factors including climate [6], seasons [7], tree species and vegetation cover [8, 9], soil features [10] and anthropic disturbance [11]. For example, in temperate forests, soil pH, tree age and precipitation have been shown to drive the assembly of fungal guilds [12]. Similarly, in grassland environments, fungal community assembly was mainly driven by available mineral nutrients and organic carbon [13] while it has been shown that plant species richness only exerts a significant influence on above-ground microbial communities [14].

For these reasons, high-throughput profiling of fungal communities has been suggested as a method to monitor forest and soil health, assuming that local fungal diversity is directly linked to ecosystem functions [15] such as litter decomposition [16] and plant-soil nutrients cycling [17].

In fungal taxonomy and community ecology, the most studied DNA barcode is the nuclear ribosomal RNA (rRNA) intergenic spacer (ITS) locus [18–20]. This fast-evolving, intron-like sequence is first transcribed as part of a large, short-lived, rRNA precursor and then sequentially eliminated during maturation of this precursor both inside and then outside of the nucleus [21–23]. The ITS can therefore be amplified using primers located within the 18S, 28S or 5.8S rRNA genes from either DNA or cDNA templates. Because of its transient nature, the (RNA) ITS whose presence is tightly linked to rDNA gene transcription, could represent a marker of cellular activity, compared to the rRNA molecules themselves that accumulate in the cytoplasm and persist in resting cells such as spores. Indeed, as RNA has a higher turnover rate compared to DNA and supposedly degrades faster than DNA following cell death, the use of environmental RNA (eRNA) instead of environmental DNA (eDNA) as a template for metabarcoding, has been advocated to better describe active microbial communities, leaving aside non-active or dead microbial cells that potentially contribute to the eDNA pool [24].

Reports of microbial, either prokaryotic or eukaryotic, community metabarcoding on both eDNA and eRNA highlighted either a strong correlation between “active” (RNA-based) and “total" (DNA-based) microbial communities [25–29], or on the contrary identified significant differences between the different datasets [28, 30, 31]. For example, a greater taxonomic alpha diversity was often, but not always (see [31]), deduced from DNA compared to RNA datasets [30–33]. This could be a consequence of the presence of DNA from dead or resting organisms (legacy DNA) that do not, or no longer, participate to ecosystem processes [34]. At the same time, several studies [30, 31, 35–37] also revealed the unexpected presence of RNA-specific taxa. As regards fungi, almost all metabarcoding studies made use of eDNA, directly extracted from environmental matrices, to characterize fungal communities after amplification and sequencing of a fungal-specific barcode sequence. Few studies reported the use of eRNA or both eDNA and eRNA to assess fungal diversity [25, 31, 37–43].

In metabarcoding studies, it is essential to identify and eliminate artefactual sequences and spurious taxa whose presence may interfere in data analysis and mask or on the contrary exacerbate differences between datasets [44]. Artefactual sequences are generated at different steps of the metabarcoding workflow, during the PCR amplification, the sequencing and the subsequent sequence assembly. A number of software have been developed to identify and remove artefactual sequences [45–48], but despite their systematic implementation many of such sequences are still present in the final MOTU files [44]. In the case of studies that generate both RNA and DNA-based datasets from the same environmental samples, it has been proposed to consider only sequences present in both datasets that define taxa shared ("shared taxa") between the two datasets [27, 28, 49]. "Shared taxa" are indeed unlikely to correspond to taxa defined on the basis of artefactual sequences and may also minimize the impact of nucleic acids from dead organisms on the make-up of microbial communities.

The aim of the present study was to compare the performance of different metabarcoding sequence datasets, generated from eDNA and eRNA, for the discrimination of fungal communities collected in different habitats at a regional scale in Northern Italy. We sampled three contrasted habitats (or substrates), namely decaying wood, forest and grassland soils. In mycology it is widely accepted, as reflected by fungal floras and field guides [32, 50, 51], that each of these habitats are characterized by specific fungal guilds and taxa, especially as far as "macrofungi" are concerned.

## Materials and methods

### Sample collection and processing

The four study sites (Table 1) are located in North-West Italy (Piedmont administrative region) and are separated from each other by between 39 to 111 km (linear geographic distances). These sites represent different climatic and biogeographic zones of this area, the continental one found at lower elevations (Mandria Regional Park, Venaria Reale), the sub-Mediterranean xeric zone (Xerotermic Oasis Protected Area of Foresto, Bussoleno) and the medium/high altitude alpine one (Pian del Creus, Chiusa di Pesio and Lombarda Pass, Vinadio). All sampled plots were located in protected areas and site selection was also based on the co-occurrence of adjacent forested and natural grassland plots of high plant biodiversity and naturalistic importance (Table 1) [52, 53]. Besides geography, climate and local vegetation, sites also differ greatly from each other with respect to geology and soil features (S1 Table). By collecting samples in these different protected undisturbed areas we therefore expected to access different, highly diverse fungal communities [6, 32, 50]. Field sampling permissions were obtained from “Parco Naturale La Mandria” and from “Parco Naturale del Marguareis” to sample respectively, in the Madria and the Creus sites. In all the other sites, no specific permissions were required. No endangered and/or protected species were involved in sampling activities.

**Table 1:** Origin and characteristics of the soil and wood samples.

Site	SCI Site Code	Collection date	Location	Coordinates	Altitude [m asl]	Mean annual temperture [°C]	Mean annual precipitation [mm]	Parental rock	Sample ID	Sample Type	Ecological features
Mandria	IT1110079	28/02/2015	Venaria Reale.TO	45°18'N 7°55'E	300	12.3	860	Quaternary sediments	MB	Forest soil ^a^	Sub—Atlantic and medio—European oak or oakhornbeam forests of the *Carpinion betuli* (9160)
									MP	Grassland soil ^a^	Molinia meadows on calcareous. peaty or clayey—siltladen soils (*Molinion caeruleae*) (6410)
									MW	Decaying wood ^b^	*Quercus robur; Carpinus betulus; Acer campestre; Corylus avellana*
Foresto	IT1110030	1/4/2015	Bussoleno.TO	45°14'N 7°10'E	500	11.4	799	Limestone	FB	Forest soil ^a^	Pannonian woods with *Quercus pubescens* (91H0)
									FP	Grassland soil ^a^	Semi—natural dry grasslands and scrublands on calcareous substrates (*Festuco—Brometalia*) (6210a)
									FW	Decaying wood ^b^	*Quercus pubescens*. *Prunus avium; Cotynus coggygria*
Creus	IT1160057	15/06/2015	Chiusa di Pesio.CN	44°20'N 7°68'E	1200	8.21	1289	Quartzites	CB	Forest soil ^a^	Acidophilous *Picea* forests of the montane to alpine levels (*Vaccinio—Piceetea*) with an *Abies alba* prevalence (9410–42.25)
									CP	Grassland soil ^a^	Mountain hay meadows (6520)
									CW	Decaying wood ^b^	*Abies alba; Fagus sylvatica; Laburnum alpinum*
Lombarda	IT1160023	18/06/2015	Vinadio.CN	44°20'N 7°14'E	2000	2.9	695	Gneiss	LB	Forest soil ^a^	Alpine *Larix decidua* forest (9420)
									LP	Grassland soil ^a^	Species—rich *Nardus* grasslands. on siliceous substrates in mountain (6230)
									LW	Decaying wood ^b^	*Larix decidua*

Geographical coordinates are expressed in WGS84 format. Ecological features describe vegetation according to [53].

^a^ soil sample.

^b^ decaying wood sample.

In both grassland and forest plots, 20 soil cores (8 cm in diameter, 15 cm in depth) were regularly collected along two distinct 20 m-long linear transects. After litter and plant removal, each sample was sieved (2 mm mesh size) and all samples from the same grassland/forest plot were mixed together in equal amounts to constitute a single composite sample that was frozen in liquid nitrogen and stored at -75°C before DNA/RNA co-extraction. About 100 pieces of decomposing wood were regularly collected in the vicinity of the two transects used for forest soil collection. Wood samples (lying on the ground or not) represented different size classes (from twigs to trunk fragments), stages of decomposition and the different tree species present on the sampling site. After removing bark fragments, wood was reduced to sawdust using a sterilized stainless steel grater. For each forest, all samples were mixed together in equal amounts, frozen in liquid nitrogen and stored at -75°C until RNA/DNA extraction.

### RNA/DNA co-extractions and RNA synthesis

Soil RNA was extracted from 2 g of material using the RNA Power Soil extraction kit from MOBIO laboratories (Carlsbad, CA, USA) according to the manufacturer’s instructions. Soil DNA was co-extracted using the PowerSoil DNA Elution kit (MOBIO laboratories). Wood RNA and DNA were co-extracted from 100 mg of wood following the protocol described by Adamo et al., [54]. Purity of the DNA and RNA extracts was evaluated by spectrophotometry (OD_260_:OD_280_ ratio, Nanodrop ND-1000 spectrophotometer, Thermo Fisher Scientific, Waltham, MA, USA) and quantified by fluorimetry using the Qubit dsDNA BR Assay kit and Qubit Fluorimeter 2.0 (Thermo Fisher Scientific).

Five hundred ng of soil/wood RNA were used for cDNA synthesis in the presence of 4 μμmol of random hexamers (10 μμl final volume). The mixture was first heated 5 min at 70°C and kept on ice for at least two min before adding 10 μμl of a reaction mixture comprising 4 μμl of a 5x buffer (Thermo Fisher Scientific); 2 μμl of a 10 μμmol dNTP solution; 1.5 μμl RNAsin RNase inhibitor at 40 U/μμl; 2 μμl of 5% Bovine Serum Albumin (BSA); 1 μμl of M-MLV Reverse Transcriptase at 200 U/μμl (Thermo Fisher Scientific) and 0.5 μμl of RNA grade water. After 1 h at 42°2°C, the enzyme was inactivated by incubating 10 min at 70°C.

### PCR amplifications and sequencing

Amplifications of fungal ITS2 sequences were performed following a nested PCR approach using as starting material either 20 ng of environmental DNA or 1 μμl of cDNA solution. The nested PCR approach was adopted to avoid the artefactual amplification of plant sequences likely to be present in the samples (soil and decaying wood), but also because the direct use of the fITS9—ITS4 primer pair on environmental nucleic acids failed to amplify the ITS2 region from several samples (see also [55, 56]). In the first PCR reaction, both the ITS1 and ITS2 (ITS1-5.8S-ITS2 DNA fragment) regions were amplified using the tagged fungal-specific primers ITS1F and ITS4 [57, 58]. In the nested PCR reaction, the tagged (unique 8 base-long tags, according to Fadrosh et al., [59]) fITS9—ITS4 primers were used [60] to amplify the shorter (ca 200–600 bp) ITS2 region suitable for Illumina sequencing and taxonomic assignation [18, 20, 60].

All amplifications were performed in a final volume of 25 μμl comprising 2.5 μμl of a 10 x Taq buffer (Thermo Fisher Scientific), 0.1 mmol dNTPs; 2 μμmol of forward and reverse primers; 0.3% of BSA; 1U of Taq DNA polymerase (Thermo Fisher Scientific); the appropriate amount of DNA or cDNA and ultrapure water. For the ITS1F—ITS4 primer pair, after an initial denaturation of 5 min at 95°C, amplification proceeded through 35 cycles of 30 s at 94°C, 45 s at 54°C and 1 min at 72°C. After a final extension for 10 min at 72°C, 1 μμl of each PCR product was used as template in the nested PCR reaction with primers fITS9 and ITS4. After an initial denaturation of 30 sec at 98°C, amplification proceeded through 30 cycles of 10 s at 98°C, 30 s at 64°C and 20 s at 72°C, followed by a 10 min extension at 72°C. All PCRs were performed in a T3000 thermal cycler (Biometra GmbH, Gottingen, Germany).

PCR products were controlled by electrophoresis on 1% agarose gels, and the four independent PCR amplifications performed on each DNA/cDNA extract were pooled in equal amounts and purified using the Wizard SV Gel and PCR Clean-Up System (Promega) following the manufacturer’s instructions. After purification the PCR products were quantified using the Qubit dsDNA BR Assay kit and Qubit Fluorimeter 2.0 (Thermo Fisher Scientific) in order to prepare libraries for a paired-end sequencing (2x250 bp) with the Illumina MiSeq technology by Fasteris (Plan-les-Ouates, Switzerland).

### Bioinformatic analyses

Bioinformatics analyses were performed essentially as described in Voyron et al., [61]. Paired-end reads were merged using PEAR v.0.9.8 [62], with quality score threshold settled at 28 and minimum read lengthioinformatics anal at 200 bp. Reads were then processed using the Quantitative Insights Into Microbial Ecology (QIIME) v.1.8 software [63]. Sequences were re-oriented when necessary, and demultiplexed based on the primer tags. Chimeric sequences were identified and removed using USEARCH61 [64], as implemented in the QIIME pipeline. Molecular operational taxonomic units (MOTUs) were determined using an open reference-based clustering strategy, with the USEARCH61 method, at a 98% sequence similarity threshold. Sequences of each demultiplexed sample of this study were deposited in the GenBank SRA under accession number SRP166716, Bioproject PRJNA498195.

Taxonomic assignments were performed with Mothur v1.35.1 and the fungal ITS UNITE database version 7.2 for Mothur [45, 65] (http://unite.ut.ee, last accessed on May 12 2017). OTU functional annotation made use essentially of the FUNGuild database [66] and integrated also other open-source data for specific taxa.

### Datasets

Starting from the bioinformatics pipeline output, two distinct datasets were created. The "all reads" dataset represented the entire original dataset (12 DNA and 12 RNA sequence datasets) from which rare MOTUs, represented by ≤ 10 reads had been removed. The "shared" datasets, encompassed reads from MOTUs identified by reads in both the DNA and RNA datasets after removal of the rare MOTUs. Prior to statistical analyses each dataset was rarefied to a common number of reads per sample using the *rrarefy* function in the R package vegan (version 2.4–3) [67]. Rarefaction thresholds of 7833 and 4239 reads were implemented for the "all reads", and "shared" datasets, respectively.

### Statistical analyses

Except when otherwise mentioned, analyses were performed in R programming environment [68] using the RStudio graphical user interface [69]. For each dataset, multivariate homogeneity of group dispersion was first assessed using the *betadisper* and *permutest* (with 1000 permutations) functions of the R package vegan. Using the same R package, one-way and two-way PERMANOVAs were performed using the function adonis. Differences in fungal communities composition among samples were visualized with a “Non-metric Multidimensional Scaling ordination” (NMDS) carried out with *metaMDS* function of the vegan R package. Differential abundance analysis was performed using the DESeq2 R package which fits a negative binomial generalized linear model to the MOTU counts table [70] using a false discovery rate (FDR) threshold of P<0.05 (Bonferroni adjusted) [71]. Differential abundance analysis was carried out on the “shared” datasets.

Comparisons between habitats were performed with the Kruskall-Wallis non-parametric test (R Base package) and the Dunn’s post-hoc test (dunn.test package v1.3.5) [72] at P<0.05. Frequencies were compared by means of the Chi-Square test, with the Pearson’s correction, when zeros were present in the frequency distributions [73]. Chi-Square test calculations were performed using WPS Office—Spreadsheets (Kingsoft Office Software).

Graphs and basic calculations were performed using WPS Office—Spreadsheets (Kingsoft Office Software) or the ggplott2 (v2.2.1) R package [74]. Ternary plots were created by means of the ggtern (v2.2.1) R package [75] and heatmap using the ComplexHeatmap package [76].

## Results

### High-throughput sequencing output and creation of different read and OTU datasets

DNA and RNA were extracted from each of the 12 decaying wood and soil samples. After reverse transcription of RNA into cDNA, fungal ITS2 was amplified from all 24 DNA and RNA (cDNA) samples. PCR products were sequenced on an IlluminaTM MiSeq system (2x250 bp reads), yielding a total of 2,390,074 (DNA) and 1,499,965 (RNA) paired-end reads. After removal of unmatched and low-quality reads, sequence clustering at a 98% sequence identity threshold produced a total of 3015 MOTUs (2404 detected in DNA and 1811 detected in RNA samples). After removal of rare MOTUs (reads ≤ 10), we identified 1345 total MOTUs that constituted the (DNA+RNA) “all reads” dataset. 913 (68.3%) of them were represented by both DNA and RNA reads and constituted the “shared" dataset, representing 88.4% of the reads.

Although removal of reads and MOTUs was performed at the level of the whole dataset (sequences of all RNA and DNA samples pooled together), for what concern the "shared" dataset, at least 75% (between 75% and 98%) of the "shared MOTUs" present in a given sample were represented by both DNA and RNA reads identified in the corresponding sample. Likewise, at least 90% (between 90% and 99%) of a sample’s reads (DNA and RNA) were affiliated to "shared MOTUs" found in the corresponding sample.

### Relationships between the DNA and RNA datasets

**Fig 1:**
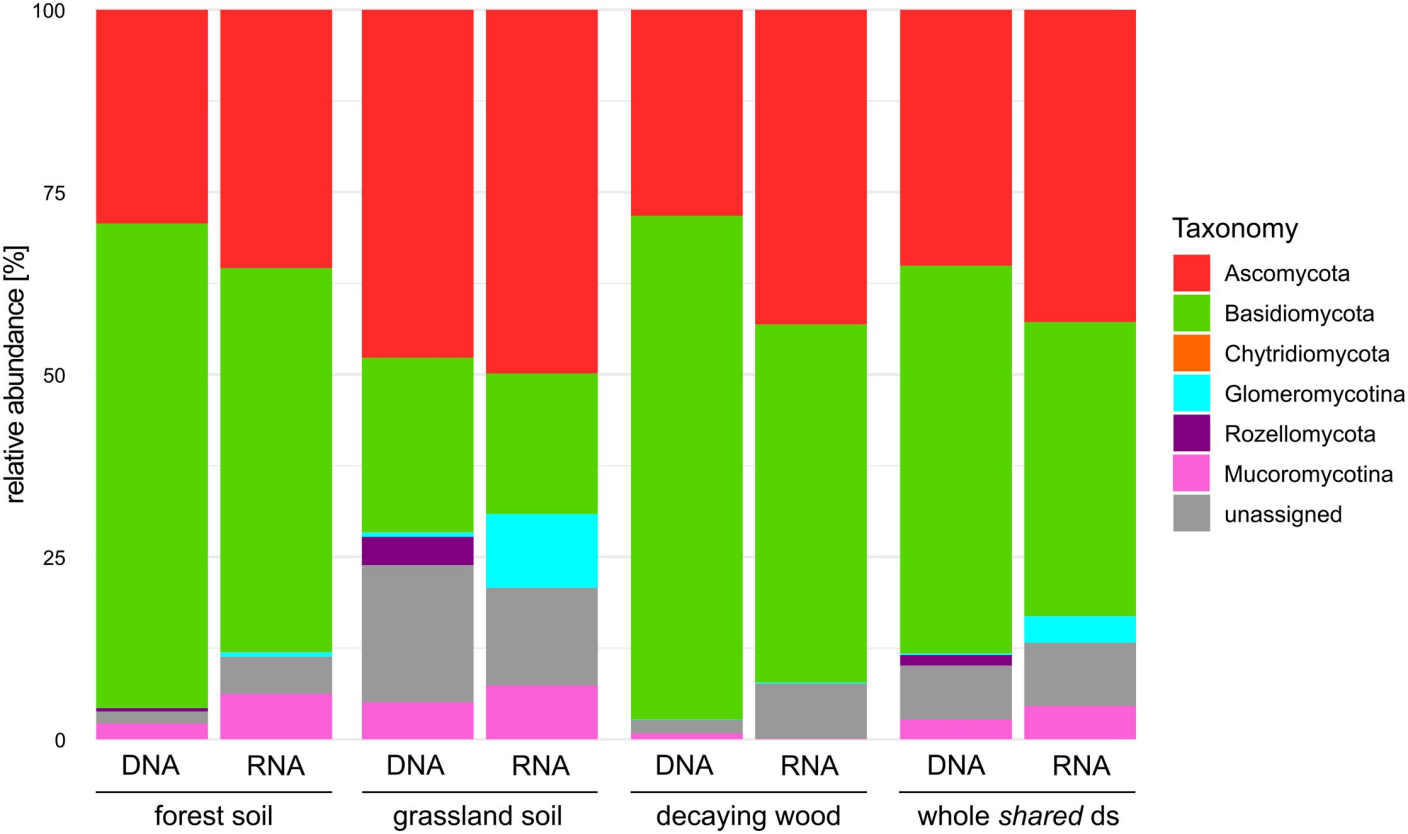
Taxon distribution (phylum or sub-phylum levels) in the eDNA and eRNA datasets. Taxon distribution is visualized for the three studied habitat, forest soil, grassland soil and decaying wood. The “whole shared ds” corresponds to the distribution in the entire dataset (all habitats together). Note the differences in abundances for Glomeromycotina and Rozellomycota between the grassland soil RNA and DNA datasets.

At a high taxonomic level (phylum and subphylum levels), fungal communities from all samples were dominated by Ascomycota and Basidiomycota taxa with minor contributions of MOTUs affiliated to the Glomeromycotina, Mucoromycotina, Chytridiomycota and Rozellomycota (Fig 1). Differences between DNA and RNA datasets were consistently observed in the different studied habitats. They regard a higher and lower proportion of reads assigned to Ascomycota and Basidiomycota respectively in the RNA datasets. The grassland ecosystem, as opposed to forest soil and decaying wood, was characterized by a significant contribution of the symbiotic Glomeromycotina to the RNA datasets (10.1% of the reads) but not to the DNA one (0.63% of the reads). For this ecosystem, an opposite trend was observed for the Rozellomycotina (3.86% of the reads in the DNA dataset compared to a complete absence in the RNA one).

**Fig 2:**
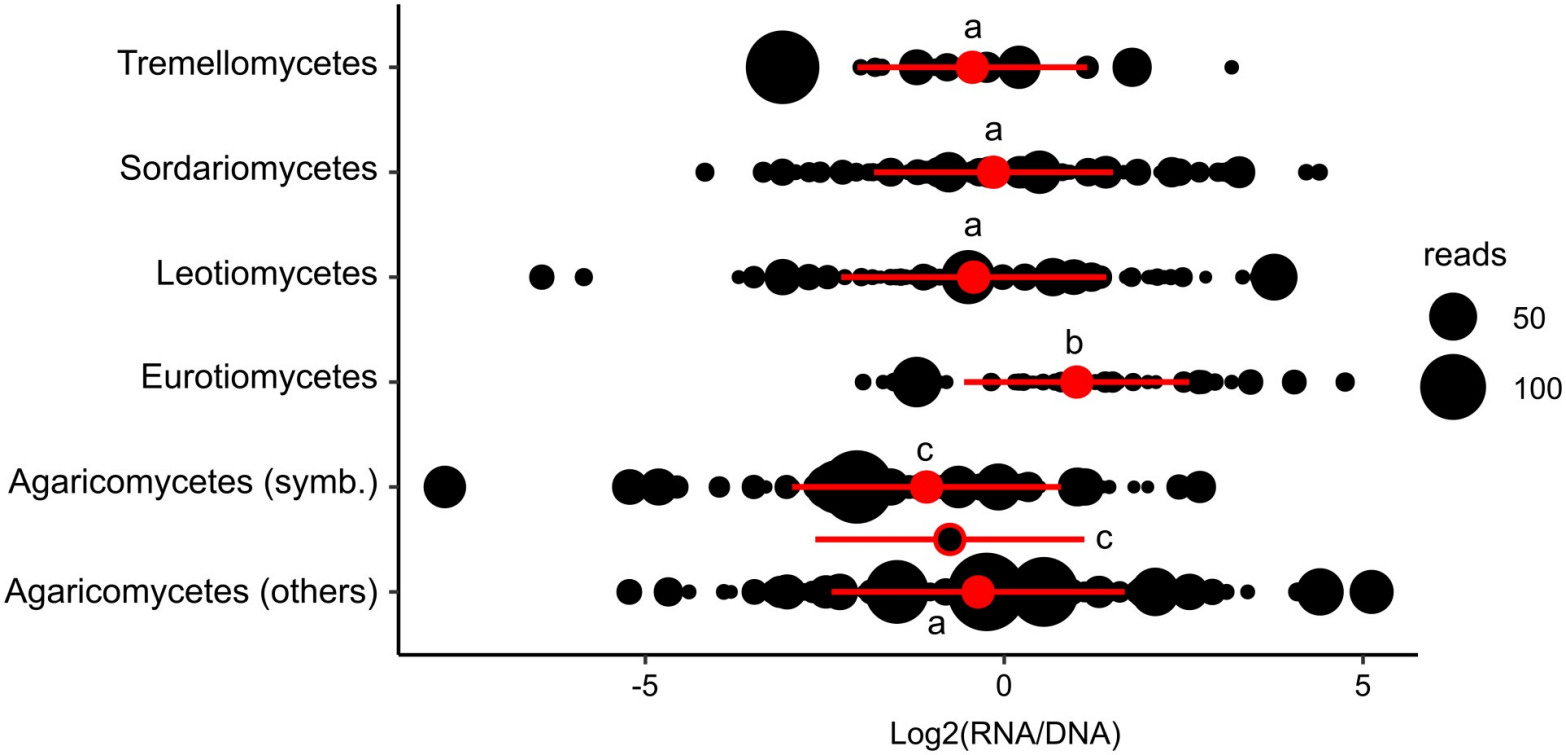
Fungal classes significantly differ from each other with respect to MOTU RNA:DNA read ratios. For each MOTU (black dots), its log_2_-transformed ratio ([No. of RNA reads]: [No. of DNA reads] +1) in the whole dataset was plotted on a horizontal axis. Symbol size is proportional to the relative abundance (average reads number among the 24 samples) of the taxa in the dataset. For Agaricomycetes we separately considered symbiotic (symb, mainly ectomycorrhizal), saprophytic and undefined (others) MOTUs. Red circles correspond to the mean values and red bars to standard deviations. In the case of Agaricomycetes, the global mean value for this class (symbiotic + non-symbiotic species together) is indicated by a black dot with red margin. Identical letters above the mean values (a, b or c) indicate which of the distributions are statistically similar (P > 0.05; Kruskall-Wallis test and Dunn’s post hoc test).

At an intermediate taxonomic level (class level), considering the five most represented classes in the "shared" dataset (with more than 15 MOTUs), different patterns of MOTU distribution were observed when considering their global RNA:DNA read ratios. Eurotiomycetes taxa, on average, were characterized by significantly higher ratios (P<0.05) compared to Sordariomycetes, Tremellomycetes and Leotiomycetes ones. By contrast, the Agaricomycetes taxa presented the lowest ratios (Fig 2). Regarding this latter class we observed that in the case of symbiotic ectomycorrhizal species there was a higher proportion (Chi^2^ test, P<0.05) of species with a negative log2 RNA:DNA read ratio compared to saprotrophic taxa (Fig 2).

**Fig 3:**
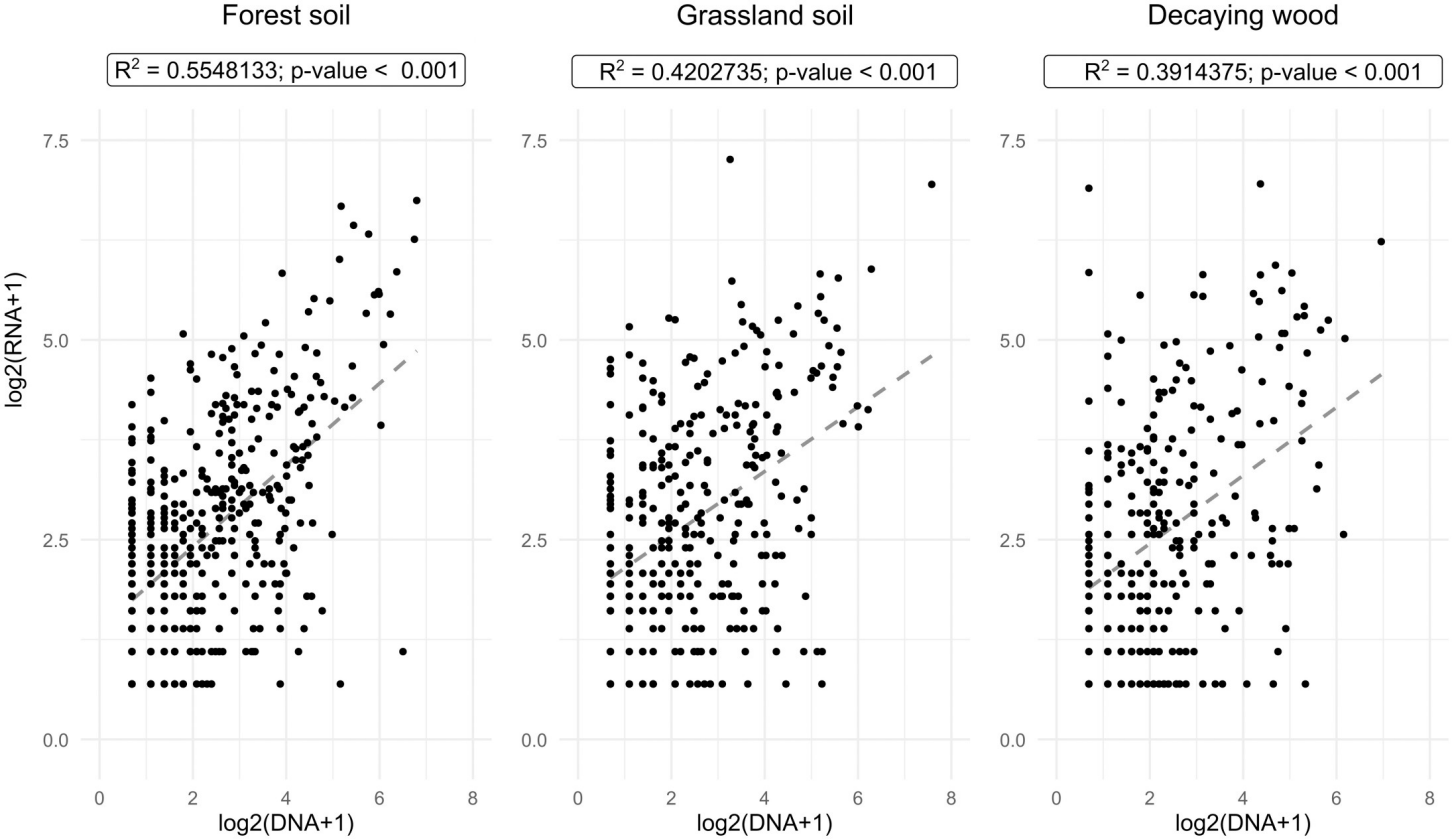
Correlations between DNA and RNA MOTUs read abundance in each of the three habitats. Log2-transformed DNA and RNA reads abundances of each MOTU are plotted against each other. Linear coefficient of correlations (R^2^) and their level of significance, as well as linear trend-lines (dashed lines) are given.

For several classes, we also observed that the distribution of taxa’s RNA:DNA read ratios differed between habitats (Kruskal-Wallis test, P<0.05) and this response was fungal class dependent (S1 Fig). For example, Tremellomycetes were enriched in the RNA fraction of decaying-wood and in the DNA fraction of grassland soil, while no specific enrichment was observed in forest soil. In the case of Sordariomycetes, an enrichment in the RNA fraction of forest soil and decaying wood was observed, and the opposite case, *i*.*e*. depletion, occurred in grassland soil. Regarding Agaricomycetes enrichment in the DNA fractions concerned both soil habitats while decaying wood showed an enrichment in the RNA fraction. Finally, at the level of individual MOTUs, considering the global dataset, MOTU relative abundances, expressed as the RNA:DNA ratios, varied significantly between MOTUs (illustrated in S2 Fig for the 30 most globally abundant MOTUs). However, although a specific MOTU could be apparently either over-, equally or under-represented in the RNA versus DNA datasets, this was not necessarily an intrinsic characteristic of the corresponding MOTU. Indeed, we observed considerable variations in the RNA:DNA ratios of many MOTUs across samples (as illustrated in S1 Fig by the bars giving the standard deviation of the values). For example, the Basidiomycota *Vuilleminia comedens* (Nees) Maire, almost equally represented as RNA and DNA reads in the global dataset (global RNA:DNA log_2_ ratio = 0.12), was either significantly over- or under-represented in the DNA or RNA datasets of the seven individual samples in which this species occurred (log_2_ RNA:DNA ratios ranging from -7.76 up to 5.35; S1 Fig). Considering each habitat separately we recorded positive, but moderate (0.39<R^2^<0.55; P<0.01) correlations between MOTUs’ relative abundances in the RNA and DNA datasets (Fig 3).

### Differences between habitats

We asked whether inclusion of DNA and RNA sequence data could modify the outcome of PERMANOVAs and pairwise comparisons between samples performed to assess the impact of habitat, considered as an environmental variable, on fungal communities. For these analyses, each of the 12 environmental samples was represented by two different sequence datasets (DNA and RNA) considered as independent (*i*.*e*. 12 environmental samples and 24 sequence datasets). One-way PERMANOVAs were thus performed on "all reads" and "shared” datasets. Both analyses highlighted significant (P<0.001) effects of both habitats and sites and their interaction on fungal community composition (S2 Table). Concerning subsequent pairwise comparisons between habitats (3 comparisons) the "all reads" dataset supported significant differences between grassland soils and wood and between forest soil and wood (P<0.05 or P<0.01), but not between grassland and forest soils (P>0.05). However, differences between forest and grassland soils were supported (P<0.05) when analyses were performed using the "shared” dataset (Table 2). These pairwise comparisons were further repeated using only the "RNA component" or the "DNA component" of the "shared” dataset. In that case, only the "RNA component", but not the "DNA component", supported differences between the grassland and forest soils (Table 2). Finally, when the analyses were done separately on the Ascomycota and Basidiomycota MOTUs represented within the "shared” dataset, significant differences were only recorded between wood and forest and between wood and grassland soils.

**Table 2:** Pairwise comparisons (two ways PERMANOVAs) between habitats using different MOTU datasets.

	Habitats pairwise comparison		
Dataset	Forest vs Grassland soil	Forest soil vs Wood	Grassland soil vs Wood
All reads	0.63	**0.003**	**0.003**
shared	**0.039**	**0.006**	**0.003**
DNA (shared) component	0.057	**0.003**	**0.003**
RNA (shared) component	**0.015**	**0.003**	**0.006**
Ascomycota	0.144	**0.006**	**0.003**
Basidiomycota	0.156	**0.003**	**0.003**

The “all reads” dataset encompasses all MOTUs with more than ten DNA and/or RNA reads, while the “shared” dataset encompasses MOTUs with more than ten reads present in both the DNA and RNA datasets. Comparisons were further performed using the DNA-only or the RNA-only component of the “shared” dataset, and also separately for the shared Basidiomycota or Ascomycota MOTUs. Analyses were performed using abundance-based Bray-Curtis indices calculated separately for each 24 datasets (3 habitats x 4 sites x 2 DNA and cDNA datasets). P—values are shown in bold when <0.05.

**Fig 4:**
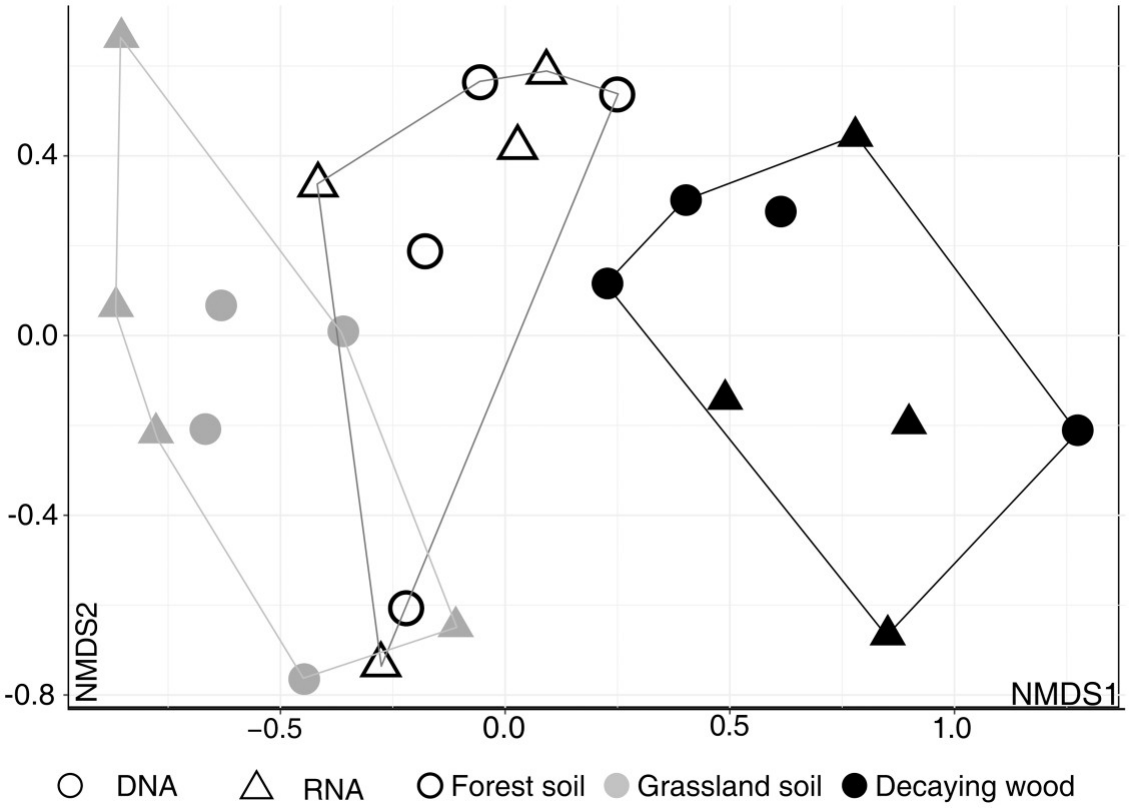
Non-Metric Multidimensional Scaling (NMDS) ordination of soil and decaying wood samples (DNA and RNA). Analysis was performed using MOTUs abundances in the “shared dataset” and Bray-Curtis indices. Convex hulls cluster samples according to habitat type. NMDS stress value = 0.048.

Differences between all three studied habitats, using the "shared” dataset, were further visualized by NMDS ordination of the 24 samples. It suggested that forest soil fungal communities occupy an intermediate position between grassland and decaying wood communities (Fig 4).

**Fig 5:**
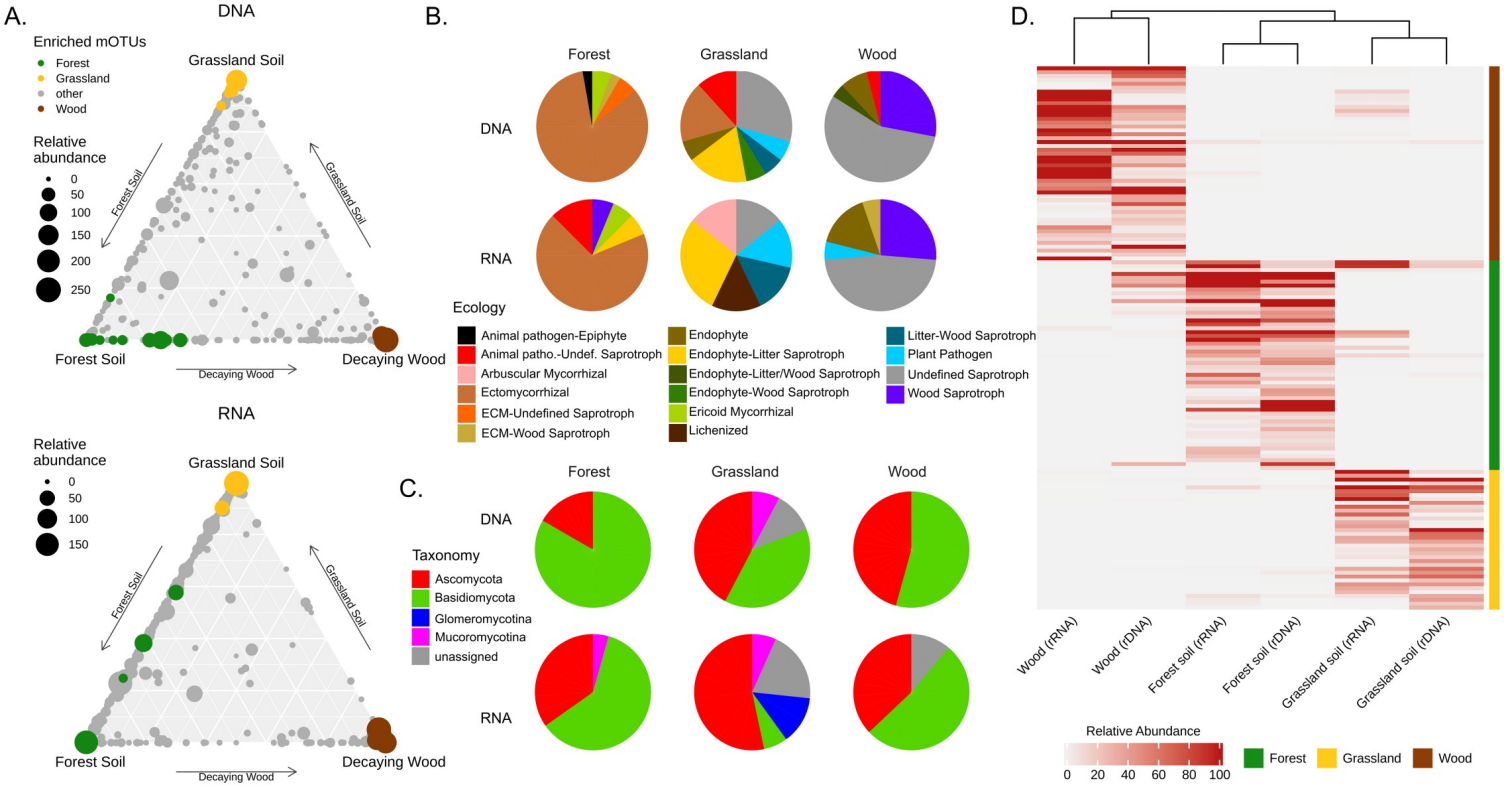
Differential abundance analysis. (A) Ternary plots illustrating the distribution of individual MOTUs (closed circles whose sizes reflect their abundance in terms or read numbers) in each of the three studied habitats. Plots were drawn separately for the DNA and RNA datasets to illustrate that the relative abundance of taxa in the three habitats varies depending on the nucleic acid used for metabarcoding. Taxa over-represented (following differential abundance analysis) in either forest soil, grassland soil or decaying wood are identified using a specific color code. (B) Pie charts that illustrate the taxonomic distribution (phylum level) and ecology (according to [Nguyen 2016 [66]]) of significantly over-represented MOTUs, identified at the species level, in each of the three habitats. (C) Mean relative abundance heatmap of enriched MOTUs in decaying wood (brown), forest soils (green) and grassland soils (yellow) across DNA and RNA samples from the different habitats. (D) Similarities between datasets was calculated using Spearman distances between samples and rows were clustered by MOTUs enriched in each habitat type. Displayed mean-relative abundance values were cut at 100 rarefied reads threshold.

The partition of MOTUs across different habitats in the “shared” dataset was visualized in ternary plots using reads abundance in either the DNA or RNA datasets (Fig 5A). Both plots showed a higher concentration of MOTUs on the grassland-woodland soil edges of the triangles, which was consistent with the observation that these two habitats are difficult to distinguish in pairwise comparisons. On the opposite, the grassland soil-decaying wood edges of the triangles were characterized by the lowest density of MOTUs. Finally, we performed differential abundance analysis to identify MOTUs enriched or depleted (P<0.05) in each of the three habitats (Fig 5A). Habitat-enriched MOTUs were then plotted on ternary diagrams with habitat-specific colors. Out of the 140 over-represented taxa identified (S3 Table), 28 were enriched in both the DNA and RNA datasets (Table 3 and Fig 5A). A dendrogram drawn using the relative abundances of enriched taxa highlighted the high similarities existing between the DNA and RNA datasets of each of the three habitats. It also evidenced a greatest proximity between the forest and grassland soils compared to decaying wood (Fig 5C).

**Table 3:** Taxonomic origin and trophic modes of the differentially abundant MOTUs identified in the three habitats.

OTU ID	Habitat	Phylum	Order	Species	Ecological guild
OTU914	Forest	Basidiomycota	Agaricales	*Amanita muscaria*	Ectomycorrhizal
OTU2401	Forest	Basidiomycota	Agaricales	*Amanita rubescens*	Ectomycorrhizal
OTU6450	Wood	Ascomycota	Helotiales	*Ascocoryne cylichnium*	Wood Saprotroph
OTU1998	Wood	Basidiomycota	Phallales	*Clathrus archeri*	Undefined Saprotroph
OTU2854	Forest	Basidiomycota	Agaricales	*Inocybe napipes*	Ectomycorrhizal
OTU11618	Grassland	Basidiomycota	Entorrhizales	*Juncorrhiza tenuis*	Plant Pathogen
OTU5201	Grassland	Ascomycota	Trapeliales	*Lambiella fuscosora*	Lichenized
OTU8369	Wood	Ascomycota	Chaetosphaeriales	*Menispora ciliata*	Endophyte
OTU6601	Wood	Ascomycota	Helotiales	*Molisia cinerea*	Wood Saprotroph
OTU32	Grassland	Zygomycota	Mortierellales	*Mortierella elongata*	Undefined Saprotroph
OTU901	Wood	Basidiomycota	Agaricales	*Mycena purpureofusca*	Leaf Saprotroph-Wood Saprotroph
OTU2272	Wood	Basidiomycota	Agaricales	*Resupinatus applicatus*	Wood Saprotroph
OTU2366	Wood	Basidiomycota	Agaricales	*Resupinatus trichotis*	Wood Saprotroph
OTU73	Forest	Basidiomycota	Russulales	*Russula atropurpurea*	Ectomycorrhizal
OTU849	Forest	Basidiomycota	Russulales	*Russula cyanoxantha*	Ectomycorrhizal
OTU782	Forest	Basidiomycota	Russulales	*Russula* sp.	Ectomycorrhizal
OTU2676	Wood	Basidiomycota	Trechisporales	*Sistotremastrum guttuliferum*	Wood Saprotroph
OTU6875	Wood	Basidiomycota	Corticiales	*Vuilleminia comedens*	Wood Saprotroph
OTU12244	Forest	Ascomycota	Leotiomycetes	NA	NA
OTU12417	Forest	Ascomycota	NA	NA	NA
OTU1340	Forest	Basidiomycota	Agaricales	NA	NA
OTU2372	Forest	Basidiomycota	Sebacinales	NA	NA
OTU3877	Forest	Basidiomycota	Agaricales	NA	NA
OTU1636	Grassland	NA	NA	NA	NA
OTU3850	Grassland	NA	NA	NA	NA
OTU3759	Wood	Ascomycota	NA	NA	NA
OTU8156	Wood	Ascomycota	Chaetosphaeriales	NA	NA
OTU1443	Wood	Basidiomycota	Agaricales	NA	NA

In this table are only listed the 28 MOTUs significantly enriched in both DNA and RNA datasets. For the complete list of the differentially abundant MOTUs see S3 Table in Supporting information. NA = not available.

Taxonomic and functional annotation of the differentially abundant taxa also underlined the specificity of each habitat (Fig 5C and S3 Table). The grassland soil habitat encompassed the highest proportion of Ascomycota (44% of the differentially abundant MOTUs) and was the only habitat to have differentially over-represented taxa belonging to the Glomeromycotina. Among Basidiomycota MOTUs over-represented in grassland samples we identified four MOTUs affiliated to *Hygrocybe*, a genus often considered as typical for this ecosystem [77]. Over-represented species in the forest soil were mostly symbiotic ectomycorrhizal species, with a major contribution of the genera *Russula* and *Inocybe* (8 MOTUs each).

In the case of Archaeorhizomycetes, a recently described class of mostly uncultivable Ascomycota reported as dominant in many type of soils [78, 79], we identified in both eDNA and eRNA samples few, low-abundance, OTUs affiliated to this class that were almost exclusively present in soil samples. Regarding the decaying wood habitat, although Basidiomycota are usually regarded as the main fungal species implicated in wood decomposition, we identified as enriched in wood samples an almost equal number of Ascomycota (47 MOTUs) and Basidiomycota (44 MOTUs) taxa.

## Discussion

The study of soil and wood-inhabiting fungal communities, conducted by their simultaneous metabarcoding on both eDNA and eRNA, highlights the complex relationship existing between these two pools of molecules. We indeed identified different factors that affect the RNA:DNA ratio of individual taxa by comparing fungal communities from three different habitats. These factors could represent confounding elements for the use of this ratio as a proxy for metabolic activity (or inactivity) of individual taxa across different environments or for comparisons between taxa. In order to better understand these relationships we compared two different datasets (“all reads” and “shared”) with the purpose to evaluate which one of them better describes fungal communities. As opposed to the “all reads” dataset that included all MOTUs, including those exclusively defined by either eDNA or eRNA reads, the “shared” dataset highlighted significant differences between grassland and forest soils that were further supported in an independent analysis based on taxa that displayed different abundances in the different studied habitats. In fungal community ecology, we therefore advice to perform, when possible, metabarcoding on both eDNA and eRNA as this approach is more likely to eliminate spurious taxa but also possibly to better reflect the true abundance of MOTUs in their respective communities. For example, we found in the “all reads” eRNA dataset a higher proportion of reads and taxa belonging to the Glomeromycota, major symbionts of herbaceous plants [80, 81], in grassland soils and of wood saprotrophs in decaying wood [82, 83]. We also identified few sequences and MOTUs affiliated to the Archaeorhizomycetes occurring almost exclusively in grassland and forest soils, thus confirming their strict association with many different types of soils [78, 79].

As in many other studies on either bacteria [27] or eukaryotic microorganisms [28, 35, 84] including fungi [29, 37, 41, 42, 85] we observed that the RNA:DNA ratio of individual MOTUs is not constant but varies between samples suggesting that local environmental factors may affect rRNA transcription levels and therefore the overall biological activity of the corresponding MOTUs. Nevertheless, we also observed that in all three studied habitats, a statistically supported global correlation between MOTU’s rRNA and rDNA read numbers existed (Fig 3), suggesting that globally, the local environmental conditions were favorable to the biological activities of most fungal taxa. Indeed, RNA- and DNA-based fungal communities do not significantly differ, as reported in other similar studies [31, 40, 42, 86] Despite this overall congruence between DNA and RNA data, we observed that MOTU’s taxonomy seemed to affect its RNA:DNA ratio (Fig 2). This deserves further studies to understand if these differences originate from taxonomically-conserved "structural features" such as, significantly higher rDNA copy number per haploid genome and/or higher densities of nuclei per volume unit of cytoplasm in the case of taxa with, on average, lower relative abundance ratios (*e*.*g*. Agaricomycetes, Leotiomycetes and Tremellomycetes). Thus far, significant differences in rDNA copy numbers per haploid genome have been reported between fungal phyla but have not been analyzed for lower taxonomic ranks (e.g. class level) [87]. We may also hypothesize that RNA:DNA sequence ratios reflect specific taxonomically-conserved trophic strategies, as suggested for bacteria [86] where low ratios were suggested to characterize oligotrophic taxa and high ratios copiotrophic ones adapted to nutrient-rich environments. In our study, it is worth noting that Eurotiomycetes MOTUs, which are characterized by the highest mean RNA:DNA ratios, encompass several fast-growing and abundantly sporulating molds, such as *Penicillium* spp. and *Aspergillus* spp. (found in our dataset), that readily colonize nutrient-rich habitats [88, 89].

In the case of fungi, although our data cannot be directly compared, a recent study by Wutkowska et al., [31] formulated a similar hypothesis for fungi, observing that symbiotrophic taxa had lower RNA:DNA ratios compared to saprotrophic ones. In our study, we also observed that among Agaricomycetes, symbiotrophic (ectomycorrhizal) species held lower log_2_(RNA:DNA) ratios compared to saprotrophic species (Fig 2). All these observations further suggest a link between RNA:DNA ITS ratios and fungal trophic strategies. In addition to soil samples we observed a similar trend in decaying wood samples. Concerning Agaricomycetes there is a notable difference in RNA:DNA ratio, that is higher in wood, dominated by saprotrophs [89–91], than in soils where mycorrhizal symbionts thrive [31, 89, 92–94].

In this study, we also showed that, although sampled were collected in contrasted geographic sites (with respect to climate, soil characteristics and vegetation), it is nevertheless possible to group fungal communities according to the substrate they originate from (grassland and forest soils, decaying wood) and that forest soil occupy an intermediate position between grassland soil and decaying wood as visualized in NMDS (Fig 4) and ternary plots analyses (Fig 5A). Proximity between forest soil and decaying wood can be explained by the fact that (i) several woody debris were collected on the ground and were probably colonized by wood/soil saprotrophs whose mycelia extend in both compartments [91, 95, 96] and (ii) several ectomycorrhizal fungi also colonize wood as a source of nitrogen [97–100].

Proximity between communities sampled in a specific habitat means that, at the studied regional scale (in North-West Italy), numerous fungal taxa specific of each of the habitats, or shared between two habitats, are widely distributed despite sharp differences in climate, vegetation and local substrate characteristics [29, 34, 101]. Differential abundance analysis identified several of these taxa and it is worth noting that several of them are among the 30 most abundant in the global sequence dataset (S2 Fig). This is the case of *Vuilleminia comedens* and *Mycena purpureofusca* (Peck)Sacc in decaying wood and of the yeast *Saitozyma podzolica* (Babeva & Reshetova) X.Z. Liu, F.Y. Bai, M. Groenew. & Boekhout, Sebacinaceae sp. and *Cenococcum* sp., in forest soils. Regarding grassland soils, we cannot exclude that the primers we used for metabarcoding the communities underestimated the occurrence and abundance of symbiotic Glomeromycotina species that do not appear in this species list [60, 81]. These widespread and abundant species may therefore represent keystone species in their respective habitats. As the genomes sequences of many of them are now available (*e*.*g*. [102–104]), this should facilitate the study of their respective contribution to ecosystem processes through metatranscriptomic or metaproteomic approaches.

## Conclusions

In conclusion, the comparison between fungal communities assessment by DNA- and RNA-based metabarcoding pointed to an overall congruence between the two methodologies. However, the combination of the two datasets highlighted differences between habitats that would have been overlooked by following an exclusive DNA-based approach. Furthermore, the combined analysis of eDNA and eRNA suggested the relative abundance of a specific MOTU in a dataset may be partly explained by its taxonomic affiliation. This observation deserves further studies to properly assess the ecological importance of specific MOTUs and taxa.

## Supporting information

S1 Table Physicochemical characteristics of the studied soil and wood samples. Soil analysis were performed by the “Laboratoire INRA d’Analyse des Soils d’Arras” (www6.hautsdefrance.inra.fr/las) using standard protocols including ISO protocols. “Volatiles” represent mass loss after combustion at 550°C. Wood lignin contents were assayed by Dr. Harald Kellner. Technical University of Dresden (D). N/A. not applicable; UN. not available.
(DOCX)

S2 Table One-Way PERMANOVAs (999 permutations) highlighting significant (P<0.01) habitat, site and site x habitat effects in each of the three main datasets commented in the study ("all reads", "10 reads" and "shared 10 reads"). (DOCX)

S3 Table Taxonomic origin and trophic modes of the differentially abundant MOTUs identified in each of the three habitats. MOTUs could be repeated if differentially abundant in one or more habitats (Habitat column) in both DNA and RNA (Library column).
(DOCX)

S1 Fig For the five most represented fungal classes, MOTUs RNA:DNA read ratios significantly differ from each other with respect to the habitat. For each MOTU (grey/orange circles) the log_2_-transformed value of the ratio [No. of RNA reads]: [No. of DNA reads] (log_2_(RNA:DNA+1)) was computed and plotted on a horizontal axis for each of the five most represented fungal classes and for habitat. Symbol size is proportional to the relative abundance (average reads number among the samples) of the taxa in the dataset. For the Agaricomycetes we distinguished symbiotic (mainly ectomycorrhizal) MOTUs (symb.) from saprotrophic and undefined ones (non-symb). Black circles give the mean values and black bars the standard deviations for each fungal class. Identical red letters on the left (a, b or c) indicate which of the distributions are statistically similar (P > 0.05; Kruskall-Wallis test and Dunn’s post hoc test).
(TIFF)

S2 Fig The 30 most abundant MOTUs (abundance defined by the absolute number of reads in the shared DNA+RNA global dataset) differ from each other with respect to their relative abundance in the "shared DNA" and "shared RNA" datasets. Log_2_ of the ([No. of RNA reads]: [No. of DNA reads] +1) ratio was calculated for each of the individual sample in which the taxon was present. Bars that give the standard deviation of the mean illustrate that for several of the taxa their relative abundance as DNA or RNA reads varied considerably between samples.
(TIFF)
