# Supplementary material for: Metabarcoding on both environmental DNA and RNA highlights differences between fungal communities sampled in different habitats
Source: PLoS One. 2020 Dec 30;15(12):e0244682. doi: 10.1371/journal.pone.0244682 (PMC7773206; doi:10.1371/journal.pone.0244682)
Supplement: S1 Table — Soil analysis were performed by the “Laboratoire INRA d’Analyse des Soils d’Arras” (www6.hautsdefrance.inra.fr/las) using standard protocols including ISO protocols. “Volatiles” represent mass loss after combustion at 550°C. Wood lignin contents were assayed by Dr. Harald Kellner. Technical University of Dresden (D). N/A. not applicable; UN. not available. (DOCX) [file pone.0244682.s001.docx]

**S1 Table**

| **Sample ID** | **Clay (< 2 m)** | **Faint silt (2/20 m)** | **Coarse silt (20/50 m)** | **Faint sand (50/200 m)** | **Coarse sand (>200 m)** | **N** | **Phosphorous  (P_2_O_5_)** | **Oragnic Carbon (C)** | **Organic matter** | **Volatiles** | **C/N** | **Cation exchange capacity** | **Calcaire (CaCO_3_)** | **pH** | **Extractives** | **Klason lignin content** | **acid - soluble lignin** |
| --- | --- | --- | --- | --- | --- | --- | --- | --- | --- | --- | --- | --- | --- | --- | --- | --- | --- |
|  | g/kg | g/kg | g/kg | g/kg | g/kg | g/kg | g/kg | g/kg | g/kg | g/100g | - | cmol_c_/kg | g/kg | - | **%** | % | % |
| MB | 197 | 295 | 267 | 167 | 74 | 2.22 | 0.017 | 33.4 | 57.7 | 9.12 | 15 | 5.55 | <1 | 4.53 | N/A | N/A | N/A |
| MP | 194 | 312 | 290 | 163 | 41 | 2.07 | 0.026 | 22.7 | 39.3 | 7.49 | 11 | 6.1 | <1 | 5.7 | N/A | N/A | N/A |
| MW | N/A | N/A | N/A | N/A | N/A | N/A | N/A | N/A | N/A | N/A | N/A | N/A | N/A | N/A | 3.15 | 33.75 | 2.93 |
| FB | 124 | 140 | 126 | 347 | 263 | 3.95 | 0.03 | 71.6 | 124 | 11.7 | 18.1 | 22.8 | 218 | 8.08 | N/A | N/A | N/A |
| FP | 117 | 139 | 143 | 413 | 188 | 3.95 | 0.02 | 71.9 | 124 | 10 | 18.2 | 18.7 | 198 | 8.14 | N/A | N/A | N/A |
| FW | N/A | N/A | N/A | N/A | N/A | N/A | N/A | N/A | N/A | N/A | N/A | N/A | N/A | N/A | UN | UN | UN |
| CB | 211 | 305 | 88 | 98 | 298 | 5.65 | 0.053 | 103 | 178 | 22.2 | 18.3 | 13.1 | <1 | 3.93 | N/A | N/A | N/A |
| CP | 200 | 267 | 109 | 114 | 310 | 5.44 | 0.073 | 56.5 | 97.8 | 13.3 | 10.4 | 8.57 | <1 | 4.72 | N/A | N/A | N/A |
| CW | N/A | N/A | N/A | N/A | N/A | N/A | N/A | N/A | N/A | N/A | N/A | N/A | N/A | N/A | 3.06 | 44.31 | 1.56 |
| LB | 269 | 247 | 82 | 118 | 284 | 5.72 | 0.053 | 87 | 150 | 20.4 | 15.2 | 11.6 | <1 | 4.81 | N/A | N/A | N/A |
| LP | 340 | 270 | 76 | 107 | 207 | 7.96 | 0.081 | 99.3 | 172 | 23.1 | 12.5 | 10.9 | <1 | 4.99 | N/A | N/A | N/A |
| LW | N/A | N/A | N/A | N/A | N/A | N/A | N/A | N/A | N/A | N/A | N/A | N/A | N/A | N/A | 4.56 | 48.02 | 0.9 |
