# Supplementary material for: Metabarcoding on both environmental DNA and RNA highlights differences between fungal communities sampled in different habitats
Source: PLoS One. 2020 Dec 30;15(12):e0244682. doi: 10.1371/journal.pone.0244682 (PMC7773206; doi:10.1371/journal.pone.0244682)
Supplement: S2 Table — (DOCX) [file pone.0244682.s002.docx]

**S2 Table**

| **all reads** | Df | SumsOfSqs | MeanSqs | F.Model | R^2^ | P |
| --- | --- | --- | --- | --- | --- | --- |
| site | 3 | 22.903 | 0.76345 | 45.052 | 0.26609 | 1,00E-04 |
| habitat | 2 | 16.863 | 0.84316 | 49.756 | 0.19591 | 1,00E-04 |
| site:habitat | 6 | 25.974 | 0.43289 | 25.545 | 0.30175 | 1,00E-04 |
| Residuals | 12 | 20.335 | 0.16946 | 0.23625 |  |  |
| Total | 23 | 86.076 | 100.000 |  |  |  |
|  |  |  |  |  |  |  |
| **shared** | Df | Sums of Sqs | MeanSqs | F.Model | R^2^ | P |
| site | 3 | 23.839 | 0.79464 | 64.255 | 0.28879 | 1,00E-04 |
| habitat | 2 | 17.689 | 0.88447 | 71.519 | 0.21429 | 1,00E-04 |
| site:habitat | 6 | 26.180 | 0.43633 | 35.282 | 0.31714 | 1,00E-04 |
| Residuals | 12 | 14.840 | 0.12367 | 0.17978 |  |  |
| Total | 23 | 82.549 | 100.000 |  |  |  |
|  |  |  |  |  |  |  |
