# Supplementary material for: Metabarcoding on both environmental DNA and RNA highlights differences between fungal communities sampled in different habitats
Source: PLoS One. 2020 Dec 30;15(12):e0244682. doi: 10.1371/journal.pone.0244682 (PMC7773206; doi:10.1371/journal.pone.0244682)
Supplement: S3 Table — MOTUs could be repeated if differentially abundant in one or more habitats (Habitat column) in both DNA and RNA (Library column). (DOCX) [file pone.0244682.s003.docx]

**S3 Table**

| **OTU ID** | **Library** | **Habitat** | **Relative abundance** | **Phylum** | **Order** | **Species** | **Ecological guild** |
| --- | --- | --- | --- | --- | --- | --- | --- |
| OTU11197 | DNA | Forest | 5.00 | Ascomycota | NA | NA | NA |
| OTU11991 | DNA | Forest | 5.17 | Ascomycota | NA | NA | NA |
| OTU12244 | DNA | Forest | 5.00 | Ascomycota | Leotiomycetes_ord_Incertae_sedis | NA | NA |
| OTU12417 | DNA | Forest | 44.00 | Ascomycota | NA | NA | NA |
| OTU6749 | DNA | Forest | 9.75 | Ascomycota | Pleosporales | *Microsphaeropsis olivacea* | Animal Pathogen-Epiphyte |
| OTU7595 | DNA | Forest | 14.08 | Ascomycota | Leotiomycetes_ord_Incertae_sedis | *Oidiodendron pilicola* | Ericoid Mycorrhizal |
| OTU9741 | DNA | Forest | 36.42 | Ascomycota | Hysteriales | NA | NA |
| OTU1007 | DNA | Forest | 3.42 | Basidiomycota | Thelephorales | NA | NA |
| OTU1194 | DNA | Forest | 6.42 | Basidiomycota | Agaricales | NA | NA |
| OTU12817 | DNA | Forest | 52.25 | Basidiomycota | Boletales | *Boletus edulis* | Ectomycorrhizal |
| OTU13 | DNA | Forest | 4.92 | Basidiomycota | Russulales | *Russula maculata* | Ectomycorrhizal |
| OTU1340 | DNA | Forest | 7.92 | Basidiomycota | Agaricales | NA | NA |
| OTU1379 | DNA | Forest | 7.67 | Basidiomycota | Thelephorales | NA | NA |
| OTU1517 | DNA | Forest | 39.67 | Basidiomycota | Sebacinales | NA | NA |
| OTU1675 | DNA | Forest | 4.67 | Basidiomycota | NA | NA | NA |
| OTU1802 | DNA | Forest | 34.83 | Basidiomycota | Atheliales | NA | NA |
| OTU184 | DNA | Forest | 10.25 | Basidiomycota | Russulales | NA | NA |
| OTU1883 | DNA | Forest | 12.00 | Basidiomycota | Cantharellales | *Hydnum repandum* | Ectomycorrhizal |
| OTU1950 | DNA | Forest | 4.50 | Basidiomycota | Agaricales | *Laccaria laccata* | Ectomycorrhizal |
| OTU2038 | DNA | Forest | 6.33 | Basidiomycota | Sebacinales | NA | NA |
| OTU2166 | DNA | Forest | 6.83 | Basidiomycota | Atheliales | NA | NA |
| OTU2372 | DNA | Forest | 48.42 | Basidiomycota | Sebacinales | NA | NA |
| OTU2401 | DNA | Forest | 14.17 | Basidiomycota | Agaricales | *Amanita rubescens* | Ectomycorrhizal |
| OTU2624 | DNA | Forest | 7.17 | Basidiomycota | Agaricales | NA | NA |
| OTU2641 | DNA | Forest | 38.42 | Basidiomycota | Atheliales | NA | NA |
| OTU2854 | DNA | Forest | 17.92 | Basidiomycota | Agaricales | *Inocybe napipes* | Ectomycorrhizal |
| OTU3576 | DNA | Forest | 4.83 | Basidiomycota | Agaricales | NA | NA |
| OTU372 | DNA | Forest | 4.75 | Basidiomycota | Boletales | *Suillus grevillei* | Ectomycorrhizal |
| OTU3776 | DNA | Forest | 3.25 | Basidiomycota | Agaricales | NA | NA |
| OTU3877 | DNA | Forest | 8.08 | Basidiomycota | Agaricales | NA | NA |
| OTU3896 | DNA | Forest | 14.50 | Basidiomycota | Agaricales | *Cortinarius alboviolaceus* | Ectomycorrhizal |
| OTU3897 | DNA | Forest | 4.58 | Basidiomycota | Agaricales | NA | NA |
| OTU3919 | DNA | Forest | 5.92 | Basidiomycota | Agaricales | *Cortinarius diasemospermus* | Ectomycorrhizal |
| OTU4068 | DNA | Forest | 25.25 | Basidiomycota | Agaricales | *Cortinarius decipiens* | Ectomycorrhizal |
| OTU593 | DNA | Forest | 15.00 | Basidiomycota | Agaricales | *Hebeloma laterinum* | Ectomycorrhizal |
| OTU726 | DNA | Forest | 13.42 | Basidiomycota | Russulales | NA | NA |
| OTU73 | DNA | Forest | 61.83 | Basidiomycota | Russulales | *Russula atropurpurea* | Ectomycorrhizal |
| OTU782 | DNA | Forest | 113.67 | Basidiomycota | Russulales | *Russula sp.* | Ectomycorrhizal |
| OTU821 | DNA | Forest | 17.08 | Basidiomycota | Agaricales | *Inocybe malenconii* | Ectomycorrhizal |
| OTU849 | DNA | Forest | 56.58 | Basidiomycota | Russulales | *Russula cyanoxantha* | Ectomycorrhizal |
| OTU914 | DNA | Forest | 26.58 | Basidiomycota | Agaricales | *Amanita muscaria* | Ectomycorrhizal |
| OTU991 | DNA | Forest | 7.00 | Basidiomycota | Cantharellales | NA | NA |
| OTU12244 | RNA | Forest | 16.25 | Ascomycota | Leotiomycetes_ord_Incertae_sedis | NA | NA |
| OTU12417 | RNA | Forest | 26.67 | Ascomycota | NA | NA | NA |
| OTU3190 | RNA | Forest | 9.08 | Ascomycota | Chaetothyriales | NA | NA |
| OTU4356 | RNA | Forest | 15.33 | Ascomycota | NA | NA | NA |
| OTU4856 | RNA | Forest | 9.75 | Ascomycota | NA | NA | NA |
| OTU6027 | RNA | Forest | 17.83 | Ascomycota | Helotiales | NA | NA |
| OTU8104 | RNA | Forest | 53.17 | Ascomycota | Capnodiales | NA | NA |
| OTU9616 | RNA | Forest | 9.08 | Ascomycota | Leotiomycetes_ord_Incertae_sedis | NA | NA |
| OTU129 | RNA | Forest | 10.50 | Basidiomycota | Cantharellales | NA | NA |
| OTU1340 | RNA | Forest | 8.67 | Basidiomycota | Agaricales | NA | NA |
| OTU1635 | RNA | Forest | 7.67 | Basidiomycota | Trechisporales | *Trechispora invisitata* | Wood Saprotroph |
| OTU1962 | RNA | Forest | 34.25 | Basidiomycota | Russulales | *Russula vesca* | Ectomycorrhizal |
| OTU2372 | RNA | Forest | 94.08 | Basidiomycota | Sebacinales | NA | NA |
| OTU2401 | RNA | Forest | 52.58 | Basidiomycota | Agaricales | *Amanita rubescens* | Ectomycorrhizal |
| OTU2854 | RNA | Forest | 10.17 | Basidiomycota | Agaricales | *Inocybe napipes* | Ectomycorrhizal |
| OTU3877 | RNA | Forest | 16.58 | Basidiomycota | Agaricales | NA | NA |
| OTU408 | RNA | Forest | 79.92 | Basidiomycota | Filobasidiales | *Solicoccozyma terricola* | Undefined Saprotroph |
| OTU4215 | RNA | Forest | 14.08 | Basidiomycota | Agaricales | *Inocybe assimilata* | Ectomycorrhizal |
| OTU73 | RNA | Forest | 46.50 | Basidiomycota | Russulales | *Russula atropurpurea* | Ectomycorrhizal |
| OTU782 | RNA | Forest | 162.25 | Basidiomycota | Russulales | *Russula sp.* | Ectomycorrhizal |
| OTU849 | RNA | Forest | 39.75 | Basidiomycota | Russulales | *Russula cyanoxantha* | Ectomycorrhizal |
| OTU914 | RNA | Forest | 78.83 | Basidiomycota | Agaricales | *Amanita muscaria* | Ectomycorrhizal |
| OTU356 | RNA | Forest | 22.92 | Zygomycota | Mortierellales | *Mortierella pseudozygospora* | Undefined Saprotroph |
| OTU4554 | DNA | Grassland | 21.17 | Ascomycota | Hypocreales | *Fusarium oxysporum* | Endophyte-Wood Saprotroph |
| OTU4566 | DNA | Grassland | 4.33 | Ascomycota | Teloschitales | NA | NA |
| OTU4650 | DNA | Grassland | 5.17 | Ascomycota | Hypocreales | *Trichoderma hamatum* | Endophyte-Wood Saprotroph |
| OTU5085 | DNA | Grassland | 12.50 | Ascomycota | Hypocreales | *Clonostachys rosea* | Plant Pathogen |
| OTU5201 | DNA | Grassland | 22.33 | Ascomycota | Trapeliales | *Lambiella fuscosora* | Lichenized |
| OTU5420 | DNA | Grassland | 7.92 | Ascomycota | Hypocreales | *Fusarium oxysporum* | Endophyte-Wood Saprotroph |
| OTU5726 | DNA | Grassland | 15.00 | Ascomycota | Pleosporales | *Pyrenochaetopsis leptospora* | Plant Pathogen |
| OTU5929 | DNA | Grassland | 6.08 | Ascomycota | NA | NA | NA |
| OTU6099 | DNA | Grassland | 13.67 | Ascomycota | Pezizales | *Picoa juniperi* | Ectomycorrhizal |
| OTU7577 | DNA | Grassland | 16.58 | Ascomycota | Sordariales | NA | NA |
| OTU8461 | DNA | Grassland | 6.25 | Ascomycota | Sordariales | NA | NA |
| OTU11618 | DNA | Grassland | 12.75 | Basidiomycota | Entorrhizales | *Entorrhiza tenuis* | Plant Pathogen |
| OTU131 | DNA | Grassland | 17.00 | Basidiomycota | Agaricales | *Hygrocybe glutinipes* | Undefined Saprotroph |
| OTU1748 | DNA | Grassland | 6.58 | Basidiomycota | Agaricales | *Hygrocybelendidissima* | Undefined Saprotroph |
| OTU1759 | DNA | Grassland | 11.33 | Basidiomycota | Agaricales | NA | NA |
| OTU2019 | DNA | Grassland | 6.83 | Basidiomycota | Agaricales | NA | NA |
| OTU2750 | DNA | Grassland | 8.75 | Basidiomycota | NA | NA | NA |
| OTU3298 | DNA | Grassland | 10.25 | Basidiomycota | Agaricales | NA | NA |
| OTU529 | DNA | Grassland | 20.92 | Basidiomycota | Agaricales | *Hygrocybe subpapillata* | Undefined Saprotroph |
| OTU543 | DNA | Grassland | 6.42 | Basidiomycota | NA | NA | NA |
| OTU772 | DNA | Grassland | 15.50 | Basidiomycota | Agaricales | *Hygrocybe chlorophana* | Undefined Saprotroph |
| OTU1636 | DNA | Grassland | 167.17 | NA | NA | NA | NA |
| OTU3850 | DNA | Grassland | 20.58 | NA | NA | NA | NA |
| OTU761 | DNA | Grassland | 37.50 | NA | NA | NA | NA |
| OTU32 | DNA | Grassland | 27.00 | Zygomycota | Mortierellales | *Mortirella elongata* | Undefined Saprotroph |
| OTU9366 | DNA | Grassland | 22.00 | Zygomycota | Mortierellales | *Mortierella rishikesha* | Undefined Saprotroph |
| OTU4636 | RNA | Grassland | 22.75 | Ascomycota | Chaetothyriales | NA | NA |
| OTU4788 | RNA | Grassland | 11.42 | Ascomycota | Venturiales | *Ochroconis icarus* | Undefined Saprotroph |
| OTU5201 | RNA | Grassland | 16.17 | Ascomycota | Trapeliales | *Lambiella fuscosora* | Lichenized |
| OTU5945 | RNA | Grassland | 12.08 | Ascomycota | NA | NA | NA |
| OTU6739 | RNA | Grassland | 38.42 | Ascomycota | Leotiomycetes_ord_Incertae_sedis | NA | NA |
| OTU7197 | RNA | Grassland | 23.75 | Ascomycota | Pezizales | NA | NA |
| OTU7502 | RNA | Grassland | 23.08 | Ascomycota | Xylariales | NA | NA |
| OTU8105 | RNA | Grassland | 13.75 | Ascomycota | NA | NA | NA |
| OTU11618 | RNA | Grassland | 11.00 | Basidiomycota | Entorrhizales | *Entorrhiza tenuis* | Plant Pathogen |
| OTU1083 | RNA | Grassland | 9.50 | Glomeromycota | Archaeosporales | NA | NA |
| OTU959 | RNA | Grassland | 182.92 | Glomeromycota | Archaeosporales | NA | NA |
| OTU1636 | RNA | Grassland | 135.00 | NA | NA | NA | NA |
| OTU3850 | RNA | Grassland | 12.08 | NA | NA | NA | NA |
| OTU4949 | RNA | Grassland | 13.83 | NA | NA | NA | NA |
| OTU32 | RNA | Grassland | 47.58 | Zygomycota | Mortierellales | *Mortirella elongata* | Undefined Saprotroph |
| OTU3759 | DNA | Wood | 12.92 | Ascomycota | NA | NA | NA |
| OTU4064 | DNA | Wood | 9.00 | Ascomycota | Saccharomycetales | *Sugiyamaella novakii* | Animal Pathogen |
| OTU4484 | DNA | Wood | 8.75 | Ascomycota | Sordariomycetidae_ord_Incertae_sedis | *Rhamphoria pyriformis* | Undefined Saprotroph |
| OTU4840 | DNA | Wood | 6.92 | Ascomycota | Xylariales | *Xylaria primorskensis* | Wood Saprotroph |
| OTU5022 | DNA | Wood | 9.83 | Ascomycota | NA | NA | NA |
| OTU6097 | DNA | Wood | 6.42 | Ascomycota | NA | NA | NA |
| OTU6302 | DNA | Wood | 12.50 | Ascomycota | Coniochaetales | NA | NA |
| OTU6374 | DNA | Wood | 7.75 | Ascomycota | Helotiales | *Bisporella citrina* | Wood Saprotroph |
| OTU6450 | DNA | Wood | 16.75 | Ascomycota | Helotiales | *Ascocoryne cylichnium* | Wood Saprotroph |
| OTU6601 | DNA | Wood | 11.92 | Ascomycota | Helotiales | *Mollisia minutella* | Wood Saprotroph |
| OTU6732 | DNA | Wood | 39.17 | Ascomycota | Leotiomycetes_ord_Incertae_sedis | NA | NA |
| OTU7138 | DNA | Wood | 17.17 | Ascomycota | Helotiales | NA | NA |
| OTU8156 | DNA | Wood | 3.25 | Ascomycota | Chaetosphaeriales | NA | NA |
| OTU8369 | DNA | Wood | 33.50 | Ascomycota | Chaetosphaeriales | *Menispora ciliata* | Endophyte |
| OTU8936 | DNA | Wood | 9.08 | Ascomycota | Chaetosphaeriales | NA | NA |
| OTU9393 | DNA | Wood | 5.92 | Ascomycota | Chaetosphaeriales | NA | NA |
| OTU1180 | DNA | Wood | 6.25 | Basidiomycota | Agaricales | *Pholiota lenta* | Litter Saprotroph |
| OTU12150 | DNA | Wood | 3.92 | Basidiomycota | Dacrymycetales | NA | NA |
| OTU1326 | DNA | Wood | 7.17 | Basidiomycota | Polyporales | *Hyphodermella rosae* | Wood Saprotroph |
| OTU1443 | DNA | Wood | 38.00 | Basidiomycota | Agaricales | NA | NA |
| OTU1872 | DNA | Wood | 22.75 | Basidiomycota | Polyporales | *Phlebia acerina* | Wood Saprotroph |
| OTU1998 | DNA | Wood | 38.83 | Basidiomycota | Phallales | *Clathrus archeri* | Undefined Saprotroph |
| OTU2010 | DNA | Wood | 25.92 | Basidiomycota | Russulales | *Stereum hirsutum* | Wood Saprotroph |
| OTU2013 | DNA | Wood | 5.00 | Basidiomycota | Polyporales | *Dichomitus campestris* | Wood Saprotroph |
| OTU2197 | DNA | Wood | 25.58 | Basidiomycota | Hymenochaetales | *Xylodon crustosus* | Wood Saprotroph |
| OTU2272 | DNA | Wood | 9.00 | Basidiomycota | Agaricales | *Resupinatus applicatus* | Wood Saprotroph |
| OTU2366 | DNA | Wood | 10.83 | Basidiomycota | Agaricales | *Resupinatus trichotis* | Wood Saprotroph |
| OTU2676 | DNA | Wood | 6.67 | Basidiomycota | Trechisporales | *Sistotremastrum guttuliferum* | Wood Saprotroph |
| OTU2813 | DNA | Wood | 11.08 | Basidiomycota | Amylocorticiales | *Amyloxenasma grisellum* | Wood Saprotroph |
| OTU3796 | DNA | Wood | 23.33 | Basidiomycota | Russulales | *Peniophora piceae* | Wood Saprotroph |
| OTU485 | DNA | Wood | 12.08 | Basidiomycota | Agaricales | NA | NA |
| OTU6052 | DNA | Wood | 4.75 | Basidiomycota | NA | NA | NA |
| OTU6875 | DNA | Wood | 252.17 | Basidiomycota | Corticiales | *Vuilleminia comedens* | Wood Saprotroph |
| OTU869 | DNA | Wood | 15.00 | Basidiomycota | Agaricales | *Megacollybia marginata* | Litter Saprotroph |
| OTU901 | DNA | Wood | 119.75 | Basidiomycota | Agaricales | *Mycena purpureofusca* | Leaf Saprotroph-Wood Saprotroph |
| OTU2847 | RNA | Wood | 12.33 | Ascomycota | NA | NA | NA |
| OTU3759 | RNA | Wood | 177.17 | Ascomycota | NA | NA | NA |
| OTU5062 | RNA | Wood | 34.67 | Ascomycota | Trichosphaeriales | *Brachysporium nigrum* | Endophyte |
| OTU5124 | RNA | Wood | 36.33 | Ascomycota | Patellariales | *Rhizodiscina lignyota* | Undefined Saprotroph |
| OTU5440 | RNA | Wood | 20.42 | Ascomycota | NA | NA | NA |
| OTU6450 | RNA | Wood | 97.25 | Ascomycota | Helotiales | *Ascocoryne cylichnium* | Wood Saprotroph |
| OTU6601 | RNA | Wood | 17.42 | Ascomycota | Helotiales | *Mollisia minutella* | Wood Saprotroph |
| OTU8156 | RNA | Wood | 16.58 | Ascomycota | Chaetosphaeriales | NA | NA |
| OTU8369 | RNA | Wood | 33.00 | Ascomycota | Chaetosphaeriales | *Menispora ciliata* | Endophyte |
| OTU9416 | RNA | Wood | 31.83 | Ascomycota | Helotiales | NA | NA |
| OTU1443 | RNA | Wood | 23.25 | Basidiomycota | Agaricales | NA | NA |
| OTU1645 | RNA | Wood | 10.92 | Basidiomycota | NA | NA | NA |
| OTU1774 | RNA | Wood | 41.42 | Basidiomycota | Trechisporales | NA | NA |
| OTU1998 | RNA | Wood | 19.50 | Basidiomycota | Phallales | *Clathrus archeri* | Undefined Saprotroph |
| OTU2272 | RNA | Wood | 104.33 | Basidiomycota | Agaricales | *Resupinatus applicatus* | Wood Saprotroph |
| OTU2366 | RNA | Wood | 20.08 | Basidiomycota | Agaricales | *Resupinatus trichotis* | Wood Saprotroph |
| OTU2499 | RNA | Wood | 114.92 | Basidiomycota | Agaricales | *Globulicium hiemale* | Wood Saprotroph |
| OTU2676 | RNA | Wood | 132.33 | Basidiomycota | Trechisporales | *Sistotremastrum guttuliferum* | Wood Saprotroph |
| OTU3105 | RNA | Wood | 10.42 | Basidiomycota | Hymenochaetales | *Hyphodontia pallidula* | Undefined Saprotroph |
| OTU4282 | RNA | Wood | 60.25 | Basidiomycota | Auriculariales | *Basidiodendron caesiocinereum* | Wood Saprotroph |
| OTU560 | RNA | Wood | 17.25 | Basidiomycota | Polyporales | *Steccherinum ochraceum* | Plant Pathogen |
| OTU622 | RNA | Wood | 44.08 | Basidiomycota | Cantharellales | *Sistotrema brinkmannii* | Wood Saprotroph |
| OTU6875 | RNA | Wood | 53.08 | Basidiomycota | Corticiales | *Vuilleminia comedens* | Wood Saprotroph |
| OTU901 | RNA | Wood | 99.33 | Basidiomycota | Agaricales | *Mycena purpureofusca* | Leaf Saprotroph-Wood Saprotroph |
| OTU10640 | RNA | Wood | 59.42 | NA | NA | NA | NA |
| OTU12449 | RNA | Wood | 50.33 | NA | NA | NA | NA |
| OTU12502 | RNA | Wood | 23.75 | NA | NA | NA | NA |
