# Supplementary figures and images for: Metabarcoding on both environmental DNA and RNA highlights differences between fungal communities sampled in different habitats
Source: PLoS One. 2020 Dec 30;15(12):e0244682. doi: 10.1371/journal.pone.0244682 (PMC7773206; doi:10.1371/journal.pone.0244682)

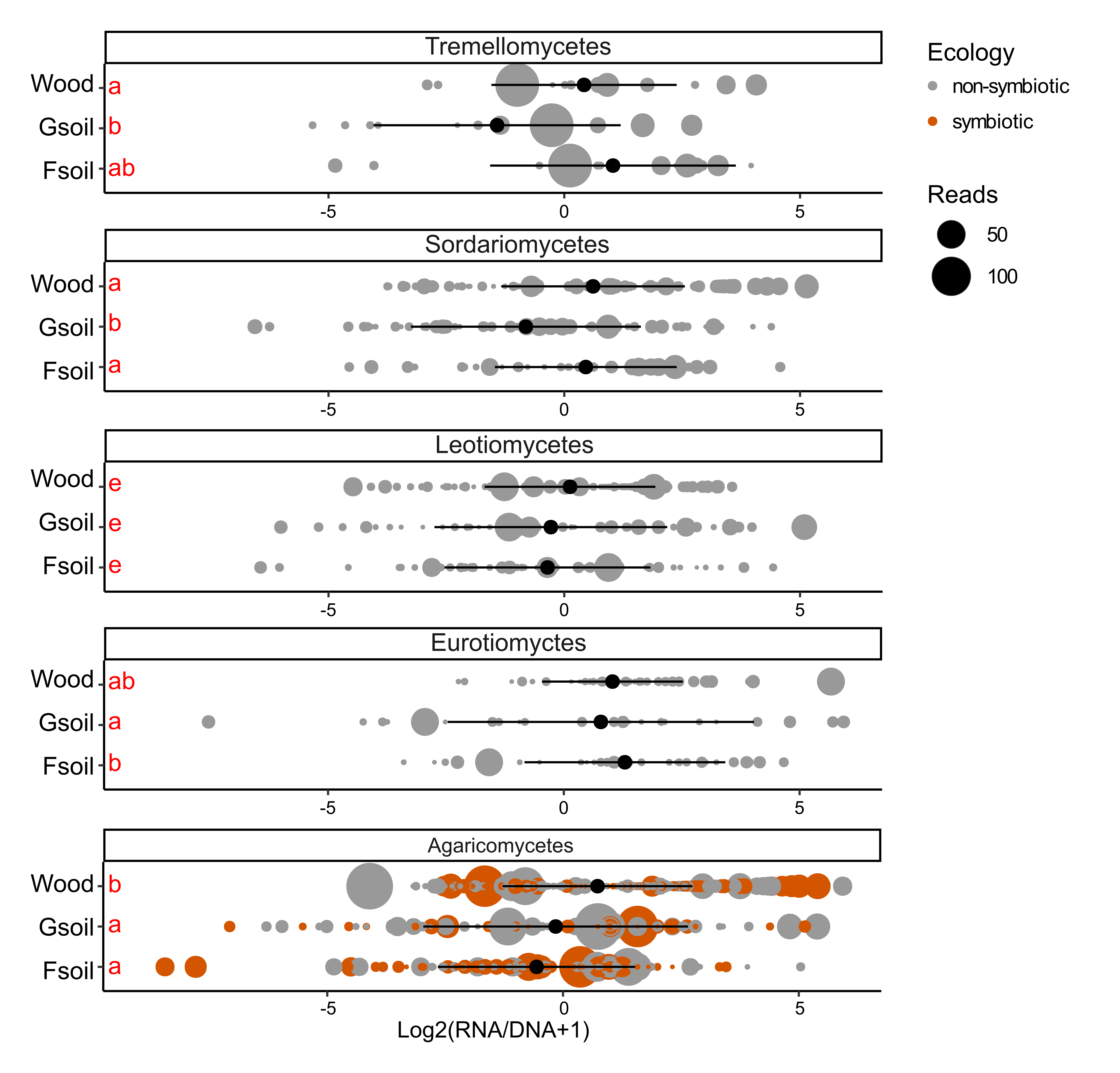

Supplement: S1 Fig — For each MOTU (grey/orange circles) the log2-transformed value of the ratio [No. of RNA reads]: [No. of DNA reads] (log2(RNA:DNA+1)) was computed and plotted on a horizontal axis for each of the five most represented fungal classes and for habitat. Symbol size is proportional to the relative abundance (average reads number among the samples) of the taxa in the dataset. For the Agaricomycetes we distinguished symbiotic (mainly ectomycorrhizal) MOTUs (symb.) from saprotrophic and undefined ones (non-symb). Black circles give the mean values and black bars the standard deviations for each fungal class. Identical red letters on the left (a, b or c) indicate which of the distributions are statistically similar (P > 0.05; Kruskall-Wallis test and Dunn’s post hoc test). (TIFF) [file pone.0244682.s004.tiff]

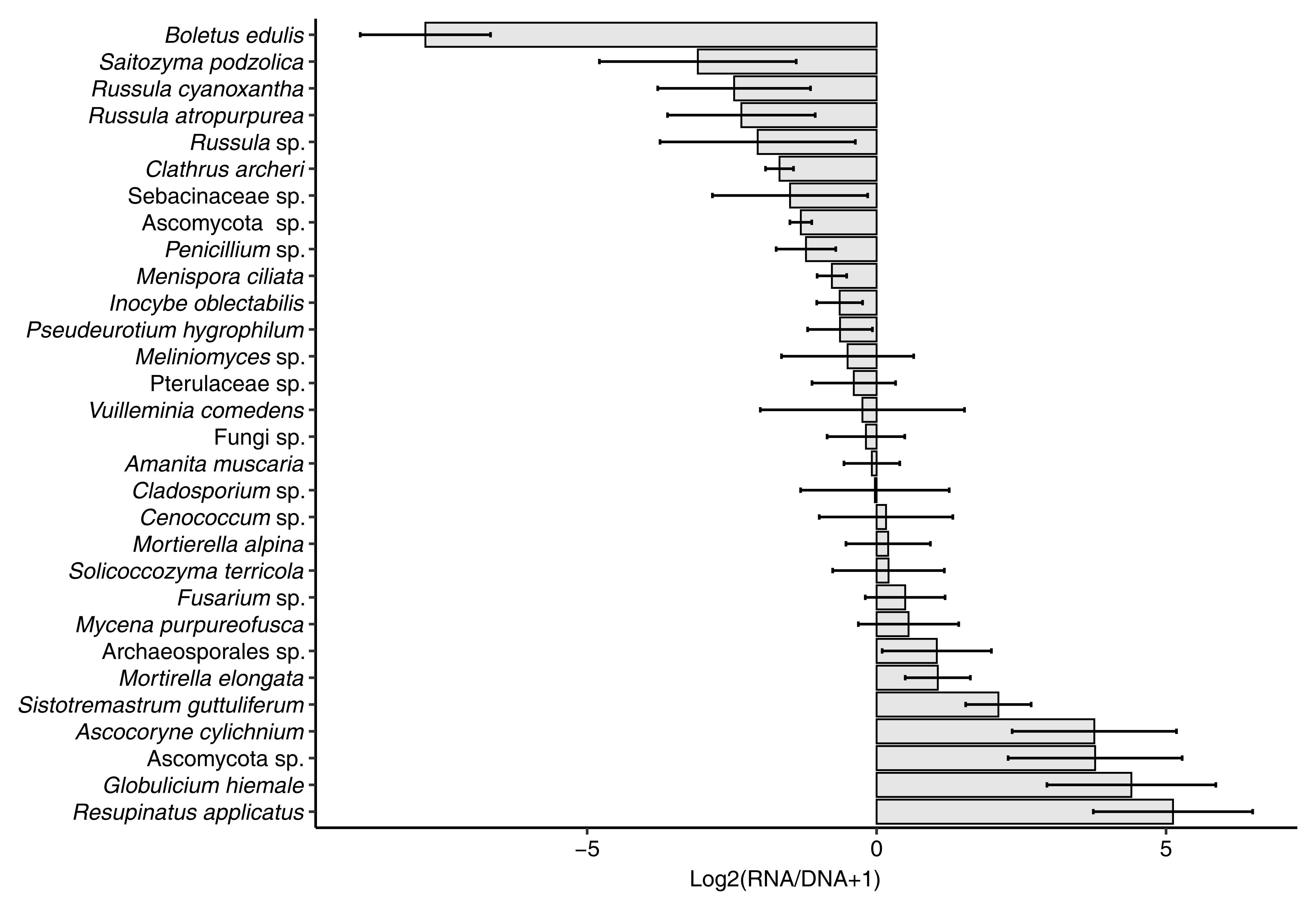

Supplement: S2 Fig — Log2 of the ([No. of RNA reads]: [No. of DNA reads] +1) ratio was calculated for each of the individual sample in which the taxon was present. Bars that give the standard deviation of the mean illustrate that for several of the taxa their relative abundance as DNA or RNA reads varied considerably between samples. (TIFF) [file pone.0244682.s005.tiff]
